# A Comparative Study of PID and Bio-Inspired Fuzzy Controllers for Speed Regulation of Low-Cost Geared DC Motors

**DOI:** 10.3390/biomimetics11090643

**Published:** 2026-09-08

**Authors:** Ionel Petrescu, Valentina-Daniela Băjenaru, Daniel-Mircea Popescu, Viorel Vulturescu, Liviu Marian Ungureanu

**Affiliations:** Department of Theory of Mechanisms and Robots, National University of Science and Technology POLITEHNICA Bucharest, 060042 Bucharest, Romania; ionel.petrescu@upb.ro (I.P.); valentina.bajenaru@upb.ro (V.-D.B.); danielmircea.popescu@upb.ro (D.-M.P.); viorel.vulturescu@upb.ro (V.V.)

**Keywords:** bio-inspired control, fuzzy logic control, PID control, DC motor speed regulation, dead-zone compensation, embedded robotics, low-cost actuators

## Abstract

Low-cost geared DC motors are widely used in mobile robotics and embedded mechatronic systems; however, their control remains challenging due to dead-zone nonlinearities, friction, low encoder resolution, motor asymmetry, and supply voltage variations. Biological organisms routinely perform motor control in the presence of similar uncertainties by relying on approximate reasoning and adaptive responses rather than precise mathematical models. This paper develops and experimentally validates a practical bio-inspired control architecture for low-cost geared DC motors operating under severe sensing and actuator limitations. The proposed controller combines fuzzy inference, dead-zone compensation, and a ramp-start mechanism to emulate the gradual and adaptive nature of biological motor responses. Instead of relying on an accurate plant model, control actions are generated through linguistic rules that mimic human-like decision-making based on speed error and error variation. The controller is implemented on an Arduino-based differential-drive robotic platform equipped with low-resolution optical encoders. Experimental results demonstrate that the proposed bio-inspired approach effectively mitigates startup stall, reduces oscillatory behavior caused by measurement quantization, and maintains stable speed regulation despite actuator variability and battery voltage fluctuations. The study shows that biologically inspired fuzzy control provides a practical and computationally efficient alternative to conventional PID methods for low-cost robotic systems characterized by significant uncertainty and nonlinear behavior.

## 1. Introduction

### 1.1. Motivation

Biological systems exhibit remarkable motor control capabilities despite operating with incomplete information, noisy sensory feedback, and continuously changing environmental conditions. Humans and animals are capable of regulating movement smoothly and efficiently without solving explicit mathematical models of the musculoskeletal system in real time. Instead, biological motor control relies on adaptive decision-making, experience-based rules, and continuous sensory feedback integration [1,2]. These characteristics have motivated the development of bio-inspired control techniques capable of handling uncertainty and nonlinear dynamics in engineering systems.

Among the various bio-inspired computational paradigms, fuzzy logic occupies a unique position because it closely resembles human reasoning processes. Rather than describing system behavior through precise mathematical equations, fuzzy control uses linguistic concepts such as “small error,” “large error,” or “speed increasing rapidly” to generate control actions. This mechanism is analogous to the way biological organisms adjust their movements based on qualitative assessments of sensory information rather than exact numerical calculations [3,4].

The relevance of bio-inspired control becomes particularly evident in low-cost robotic platforms. Unlike industrial servo-systems, inexpensive geared DC motors exhibit strong nonlinear behavior, including static friction, dead-zone effects, gearbox backlash, parameter variability, and sensitivity to battery voltage changes. Furthermore, low-resolution encoders provide quantized speed measurements that introduce uncertainty into the feedback loop. These limitations make accurate mathematical modeling difficult and often reduce the effectiveness of conventional model-based control techniques.

Classical PID controllers remain the dominant solution for DC motor speed regulation because of their simplicity and ease of implementation. Nevertheless, their performance is strongly dependent on parameter tuning and often requires additional mechanisms such as anti-windup protection, filtering, and dead-zone compensation. In practical low-cost robotic systems, these requirements may increase implementation complexity while still providing limited robustness against changing operating conditions [1].

### 1.2. Related Work

Inspired by biological motor adaptation, this work investigates a fuzzy logic speed controller specifically designed for low-cost geared DC motors operating under limited sensing capabilities. The controller combines biologically motivated approximate reasoning with two practical mechanisms: dead-zone compensation and ramp-start control. The dead-zone compensation addresses static friction effects that prevent motion initiation, while the ramp-start mechanism imitates the gradual force application commonly observed in biological locomotion, thereby reducing abrupt accelerations and startup oscillations [5].

The proposed approach is experimentally validated on a differential-drive robotic platform using low-resolution optical encoders and an Arduino-compatible microcontroller. The objective is not to replicate biological motion directly, but rather to exploit biological principles of adaptive and robust decision-making to improve motor speed regulation in uncertain and resource-constrained environments.

Furthermore, advance brain-inspired robotic control strategies emphasize the integration of sensory feedback, adaptive responses, and biologically motivated decision-making mechanisms to improve robot performance under uncertain operating conditions [6].

### 1.3. Biological Motivation

Biological organisms perform motor control in environments characterized by uncertainty, disturbances, and incomplete sensory information. Human and animal locomotion, for example, relies on the continuous integration of sensory feedback from vision, proprioception, and tactile receptors to regulate movement and maintain stability. Rather than depending on precise mathematical models of muscles, joints, and environmental interactions, biological systems employ adaptive mechanisms that generate appropriate responses based on qualitative assessments of the current state [7].

Research in neuroscience has shown that motor control is achieved through hierarchical processes involving feedback correction, sensory integration, and learning-based adaptation. When a deviation from the intended movement is detected, corrective actions are not computed through exact analytical equations but are generated through distributed neural mechanisms that continuously adjust motor commands according to the magnitude and direction of the perceived error. This behavior enables biological systems to remain functional despite sensor noise, changing loads, and variations in environmental conditions [7,8].

The challenges encountered in low-cost robotic systems share several similarities with those faced by biological motor systems. Inexpensive DC motors often operate under uncertain conditions caused by friction, actuator variability, dead-zone nonlinearities, battery voltage fluctuations, and noisy measurements from low-resolution sensors. These factors complicate the development of accurate mathematical models and can significantly degrade the performance of conventional model-based controllers.

Fuzzy logic control provides a computational framework that reflects several characteristics of biological decision-making. Instead of requiring an exact plant model, control actions are derived from linguistic rules that encode expert knowledge in a form analogous to qualitative human reasoning. Concepts such as “if the speed is much lower than desired, increase the motor command significantly” or “if the speed is close to the target, apply only a small correction” resemble the heuristic strategies employed by biological organisms when regulating movement [9,10,11]. Previous research has shown that fuzzy-PD control can provide an efficient and affordable solution for low-cost robotic platforms characterized by nonlinear dynamics and limited hardware resources [12].

The proposed controller incorporates additional mechanisms inspired by biological behavior. The dead-zone compensation can be interpreted as an artificial analog of the minimum muscular activation required to overcome static friction and initiate movement. Similarly, the ramp-start mechanism imitates the gradual force generation observed during biological locomotion, where acceleration is progressively increased rather than applied instantaneously. Together, these mechanisms enable smoother motion initiation and improved adaptation to the nonlinear characteristics of low-cost actuators.

Although the proposed controller does not attempt to replicate the physiological structure of biological motor systems, it adopts key bio-inspired principles, including approximate reasoning, feedback-driven adaptation, robustness to uncertainty, and gradual response generation. These characteristics make fuzzy control particularly suitable for embedded robotic applications where sensing capabilities, computational resources, and actuator precision are inherently limited.

**Figure 1:**
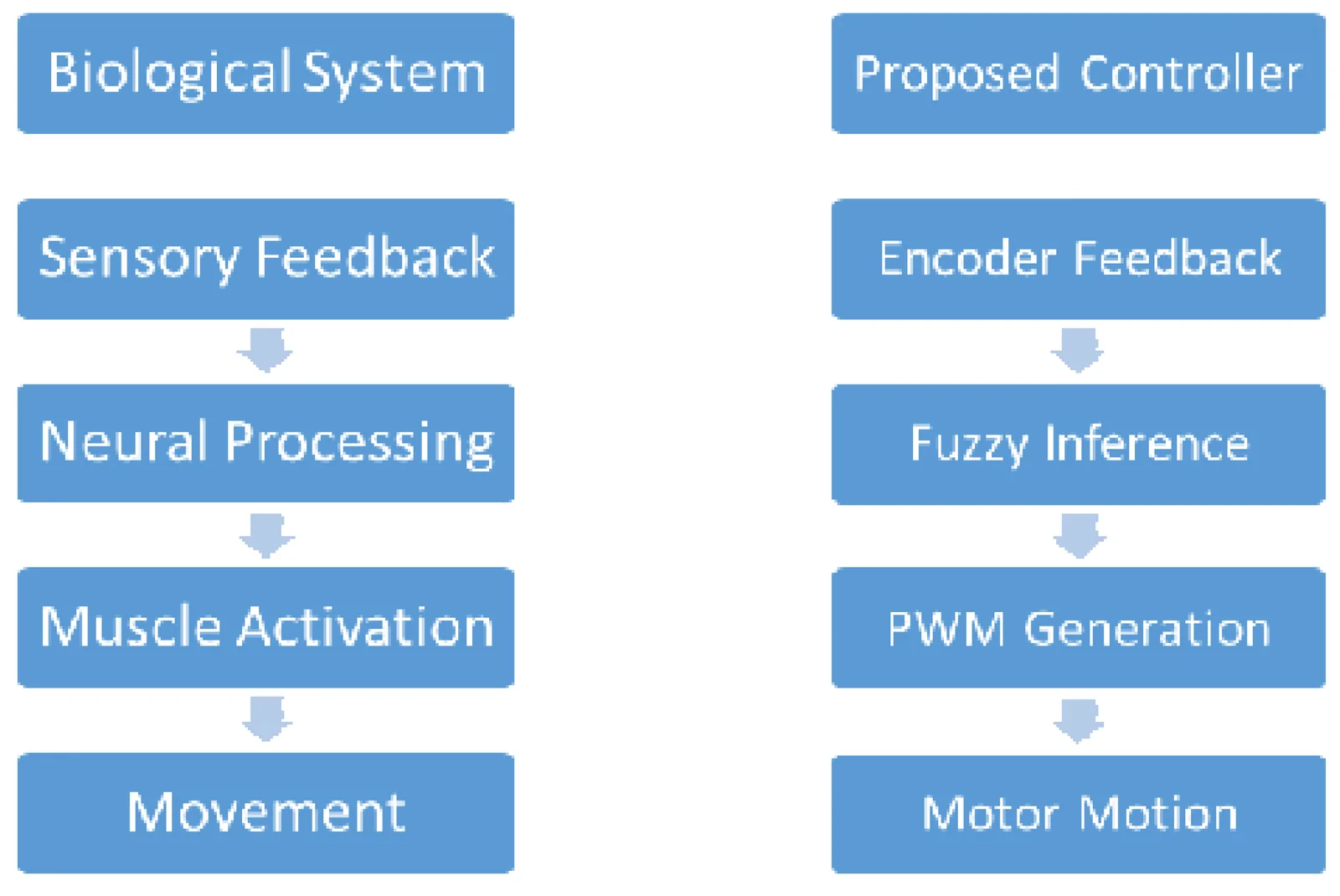
Comparison between biological motor regulation and the proposed bio-inspired fuzzy control architecture. In both systems, corrective actions are generated from feedback information through adaptive decision-making mechanisms rather than through explicit mathematical inversion of the controlled system.

Figure 1 shows a conceptual comparison between biological motor control and the proposed bio-inspired fuzzy controller.

#### Biological Interpretation of the Proposed Controller

The proposed controller does not attempt to reproduce the physiological structure of the human nervous system. Instead, it adopts several control principles that are commonly observed in biological motor regulation.

First, the fuzzy inference mechanism replaces an explicit mathematical model with qualitative decision-making based on linguistic rules. This resembles biological motor control, where corrective actions are generated from sensory perception and experience rather than analytical computation.

Second, the controller continuously adjusts the motor command using sensory feedback obtained from the optical encoder. Similarly, biological motor systems continuously modify muscle activation according to proprioceptive and sensory feedback to maintain the desired movement despite disturbances.

Third, dead-zone compensation can be interpreted as an analog of the minimum muscle activation required to overcome static friction and initiate movement. In biological systems, muscles must first generate sufficient force before limb motion begins.

Finally, the ramp-start mechanism mimics the gradual increase in muscle activation observed during voluntary movement. Rather than applying maximum torque instantaneously, biological systems progressively recruit motor units, producing smooth acceleration and reducing mechanical stress.

Although simplified, these mechanisms incorporate biologically inspired principles of adaptive feedback regulation, gradual force generation, and approximate reasoning into a computationally efficient controller suitable for embedded robotic systems.

### 1.4. Novelty and Main Contributions

Although fuzzy logic and PID control have been extensively investigated for DC motor speed regulation, most published studies assume relatively linear actuator characteristics, high-resolution sensing, or simulation-based validation. In contrast, low-cost geared DC motors used in educational and service robots exhibit pronounced nonlinearities, including dead-zone behavior, gearbox friction, motor-to-motor variability, encoder quantization, and supply voltage fluctuations. These practical limitations are rarely addressed simultaneously within a single control framework.

The novelty of this work does not reside in proposing a new fuzzy inference algorithm. Instead, it lies in developing and experimentally validating a bio-inspired control architecture specifically tailored for low-cost embedded robotic platforms operating under severe hardware constraints.

The scientific novelty of this research does not reside in the development of an isolated fuzzy inference algorithm. Instead, it lies in the structural synthesis and experimental validation of a unified bio-inspired embedded architecture that simultaneously counteracts severe, concurrent hardware limitations—such as nonlinear dead zones, severe encoder quantization noise, and low-cost actuator asymmetry—without relying on analytical plant modeling. The core contributions of this work are precisely summarized into three key pillars:

Co-Design of a Unified Bio-Inspired Framework: Synthesis of an incremental fuzzy engine coupled with an analytical dead-zone compensator and a gradual ramp-start layer, mathematically emulating biological motor unit recruitment to stabilize highly uncertain, nonlinear electromechanical plants.

Quantification of Control Smoothness Trade-offs: An experimental demonstration that biologically inspired approximate reasoning drastically reduces high-frequency control chattering—achieving a 56% decrease in Total Variation (TV)—effectively trading aggressive linear rise times for long-term electromechanical lifespan preservation.

Resource-Constrained Resource Validation: Successful low-overhead deployment of a high-dimensional 7 × 7 rule-base fuzzy control loop on an industrial 8-bit Arduino microcontroller, providing a deterministic, mathematically sound alternative to PID control for low-cost educational and service robotics operating under extreme battery and sensory limitations.

## 2. System Modeling and Control Design

### 2.1. DC Motor Modeling

The speed dynamics of a brushed DC motor can be described by the well-known electrical and mechanical equations relating armature voltage, current, torque, and angular velocity. These models provide useful insight into the motor behavior and form the basis of many classical control approaches. However, for inexpensive geared DC motors, the actual system exhibits significant nonlinearities, including static friction, gearbox backlash, dead-zone effects, motor-to-motor variability, and supply voltage fluctuations. These factors make accurate mathematical modeling difficult and motivate the use of model-independent control strategies such as fuzzy logic.

Although the classical DC motor model is useful for understanding the underlying dynamics, the proposed controller does not rely on an accurate mathematical model. Instead, it employs a bio-inspired fuzzy control strategy capable of accommodating the nonlinear behavior commonly encountered in low-cost geared DC motors.

### 2.2. PID Controller Design

The PID controller is implemented in a closed-loop configuration, where the speed error is defined as follows:

(1) $$e\left(t\right)=\omega_{ref}\left(t\right)-\omega (t)$$

The PID control law is the following:

(2) $$u\left(t\right)=K_{p}e\left(t\right)+K_{i}\int_{0}^{t}e\left(t\right)dt+K_{d}\frac{de(t)}{dt}$$

where

- (Kp), (Ki), (Kd)—proportional, integral, and derivative gains, respectively
- (u(t))—control voltage applied to the motor

The gains are tuned using classical methods such as Ziegler–Nichols’ trial and error or Cohen–Coon tuning [13,14,15]. The derivative term improves damping and transient response, while the integral term eliminates steady-state error but may introduce integral windup under actuator saturation [16]. The objective is to minimize rise time, overshoot, and steady-state error while maintaining stability. In this study, the PID controller is implemented on an Arduino using direct computation of the control law, with PWM output applied to the motor driver.

### 2.3. Fuzzy Logic Controller Design

The Fuzzy Logic Controller (FLC) is designed to emulate human reasoning in adjusting motor speed. Unlike PID, the FLC does not require an exact mathematical model and relies on linguistic rules based on expert knowledge [17,18]. Comparative studies of DC motor drive systems have shown that fuzzy logic controllers can provide improved robustness and smoother responses under nonlinear operating conditions, whereas PID controllers generally offer faster transient performance when accurately tuned [19].

The Fuzzy Logic Controller (FLC) provides an intelligent alternative to classical PID control for DC motor speed regulation. It handles nonlinearities, load variations, and system uncertainties without requiring an exact mathematical model. 

#### 2.3.1. Inputs and Output

Error (e)—the difference between the reference speed and actual speed is as follows:

(3) $$e=\omega_{ref}-\omega$$

Change in Error (Δe)—the difference between consecutive errors is as follows:

(4) e=e(t)−e(t−1)

#### 2.3.2. Membership Functions

Triangular membership functions are calculated as follows:

(5) $$\mu_{a}\left(x\right)=\{\begin{array}{c} 0,\;\;\;x\leq a\;or\;x\geq c\\ \frac{x-a}{b-a},\;a<x<b\;\;\\ \frac{c-x}{c-b},\;\;\;b\leq x<c \end{array}$$

Trapezoidal membership functions are calculated as follows:

(6) $$\mu_{a\;}\left(x\right)=\{\begin{array}{c} \begin{array}{c} 0,x\leq a\;\;\\ \frac{x-a}{b-a},\;a<x\leq b\\ 1,b<x\leq c \end{array}\\ \frac{d-x}{d-c},c<x\leq d\\ 0,x>d \end{array}$$

#### 2.3.3. Rule Base Configuration

The linguistic set of seven variables is as follows:

- NB = Negative Big;
- NM = Negative Medium;
- NS = Negative Small;
- ZE = Zero;
- PS = Positive Small;
- PM = Positive Medium;
- PB = Positive Big.

#### 2.3.4. Rule Table

The complete 7 × 7 fuzzy rule base is presented in Table 1.

#### 2.3.5. Inference

We used the Mamdani method [13,14,15]. Triangular and trapezoidal membership functions are widely used in embedded fuzzy controllers because of their low computational complexity and efficient real-time implementation:

(7) $$\mu_{U}\left(u\right)=\underset{i,j}{\max}\left[min(T\left(\mu_{Ai}\left(E\right),\mu_{Bj}\left(DE\right)\right),\mu_{C_{u(i,j)}}\left(u\right))\right]$$

The mathematical symbols and fuzzy-control terms are defined in Table 2.

#### 2.3.6. Implementation of Centroid Defuzzification Method 

The centroid method is used to convert fuzzy outputs into a crisp PWM value, which is then applied to the motor driver [13,14,15]:

(8) $$u=\frac{\sum_{i=1}^{n}\mu_{i}x_{i}}{\sum_{i=1}^{n}\mu_{i}}$$

$x_{i}$ = representative output value of rule iii;

$\mu_{i}$ = membership degree of rule iii;

*n* = total number of active rules.

Advantages of the Fuzzy Logic Controller are as follows:

- Handles nonlinearities and parameter variations better than PID.
- Provides smoother control with reduced overshoot and oscillations.
- Does not require an exact mathematical model.

This fuzzy approach allows the controller to adapt to nonlinearities and load variations, providing smoother and more robust speed regulation compared to classical PID control.

#### 2.3.7. Quantitative Formulation of the Fuzzy Controller

To provide a rigorous mathematical foundation for the implemented control loop and address continuous-to-discrete mapping, the proposed Fuzzy Logic Control subsystem can be analytically formalized as a deterministic nonlinear function. Let the continuous state-space vector of the error tracking at any sampling instant *k* be defined as follows:

(9) $$x\left(k\right)={\left[e\left(k\right),\Delta e\left(k\right)\right]}^{T}\in R^{2}$$

The nonlinear input scaling mechanism transforms the physical state variables into a normalized compact domain, X=[−1,1]x[−1,1], via a strictly bounded linear projection operator sat(·).

(10) $$E\left(k\right)=max\left(-1,min\left(1,\frac{e(k)}{e_{max}}\right)\right)$$

(11) $$\Delta E\left(k\right)=max\left(-1,min\left(1,\frac{\Delta e(k)}{\Delta e_{max}}\right)\right)$$

Let $A_{i}$ and $B_{j}$ (where i,j∈{1,2,…7} denote the linguistic fuzzy sets defining the partitions of the input universes, *E* and Δ*E*, respectively. The exact algebraic mapping of the triangular membership profiles evaluated for a generic scalar operand X∈{E,ΔE} parameterized by the structural support coordinates $\left[a_{i},b_{i},c_{i}\right]$ is rigorously formulated as follows:

(12) $$\mu_{A_{i}}\left(X\right)=max\left(0,min\left(\frac{X-a_{i}}{b_{i}-a_{i}},\frac{c_{i}-X}{c_{i}-b_{i}}\right)\right)$$

The operational firing strength $\alpha_{i,j}\left(k\right)$ inside the 7 × 7 linguistic relational rule matrix is governed by the standard intersection operator (Mamdani minimum T-norm):

(13) $$\alpha_{i,j}\left(k\right)=T\left(\mu_{A_{i}}\left(E\left(k\right)\right),\mu_{B_{j}}\left(\Delta E\left(k\right)\right)\right)=min\left(\mu_{A_{i}}\left(E\left(k\right)\right),\mu_{B_{j}}\left(\Delta E\left(k\right)\right)\right)$$

The multi-dimensional aggregation of individual rule contributions mapping into a specific output fuzzy term $C_{k}$ is resolved using the maximum S-norm operator across the active coordinate subspace $\Omega_{k}$:

(14) $$\mu_{{\Delta U}_{k}}\left(u\right)=\underset{\left(i,j\right)\epsilon \Omega_{k}}{\max}\left[\alpha_{i,j}\left(k\right)\right]$$

(15) $$\Omega_{k}=\left\{\left(i,j\right)|Rule\left(i,j\right)\to C_{k}\right\}$$

The analytical defuzzification via the center-of-gravity (centroid) formulation yields the exact non-dimensional control increment ΔU(k) ϵ [−1,1]:

(16) $$\Delta U\left(k\right)=\frac{\sum_{k=1}^{7}\mu_{\Delta U_{k}}*\zeta_{k}}{\sum_{k=1}^{7}\mu_{\Delta U_{k}}}$$

where $\zeta_{k}$ represents the localized geometric center-point of the output membership function distribution $C_{k}$. The non-dimensional control increment ΔU(k) is mapped into the physical domain via a linear scaling factor $\Delta u_{max}$:

(17) $$\Delta u\left(k\right)=\Delta U\left(k\right)\cdot \Delta u_{max}$$

Finally, the real-time physical command applied to the L298 H-bridge motor driver at step k is calculated sequentially through discrete integration (accumulation) of the control increments, subject to anti-windup saturation constraints:

(18) $$u\left(k\right)={sat}_{0}^{255}\left(u\left(k-1\right)+\Delta u\left(k\right)\right)$$

This quantitative framework explicitly describes the mathematical continuity of the controller, defining a deterministic piecewise nonlinear surface $f:R^{2}\to R$ where Δu(k)=f(e(k),Δe(k)), eliminating empirical ambiguities.

## 3. Practical Implementation and Experimental Results

### 3.1. Hardware Setup and Experimental Methodology

For practical implementation, we used a two-wheel drive robot. To study the effectiveness of the controllers, we intentionally chose low-capability motors with a max speed of 260 RPM at 6 V and 120 mA, a gear ratio of 1:48, and a torque of 0.8 kg * cm. From construction, they had different frictions, gear backlashes, encoder alignments, supply voltages at the driver, dead zones, gear efficiency, and torque constants.

The controllers were then implemented on an Arduino Uno, driving a small DC motor through an H-bridge L298, and motor speed was measured using an IR sensor with 20 encoder slots [12,20,21].

The experiments focused on forward motion only, allowing independent speed regulation of the left and right motors. A sampling period of 100 ms was adopted for the fuzzy controller, while the encoder measurements were filtered using an exponential moving average (EMA) with a smoothing coefficient of α = 0.1 to reduce noise quantization associated with the low encoder resolution. Previous experimental tuning showed that this value provided the best compromise between measurement stability and controller responsiveness.

The target speed was fixed at 80 RPM, corresponding to approximately 83% of the nominal no-load motor speed (96 RPM). This operating point was selected because preliminary experiments indicated that it provides reliable operation while avoiding saturation effects frequently observed near the maximum motor speed.

The fuzzy controller employed seven triangular membership functions for both the speed error and the variation in the speed error, resulting in a 7 × 7 rule base. The controller parameters were experimentally determined using repeated trial-and-error tuning to obtain stable operation on the investigated motor platform. The final scaling factors were as follows:

Maximum speed error: emax = 40 RPM

Maximum error variation: Δemax = 200 RPM/s

Maximum control increment: Δumax = 15 PWM counts

Because of manufacturing tolerances between the two inexpensive motors, different base PWM values were required to achieve similar steady-state speeds. The final values were 70 PWM counts for the left motor, and 73 PWM counts for the right motor.

To improve startup reliability, two additional mechanisms were incorporated into the controller. Dead-zone compensation was introduced to overcome static friction and gearbox resistance commonly observed in inexpensive geared motors, thereby preventing startup stall. In addition, a ramp-start mechanism gradually increased the motor command during the initial acceleration phase to reduce abrupt PWM variations and smooth the transition toward the desired operating speed.

The proposed controller was experimentally compared with a conventionally tuned PID controller implemented on the same hardware platform under identical operating conditions. The PID parameters were obtained through experimental tuning and represented the best compromise between tracking accuracy and stability for the investigated motors.

Controller performance was evaluated using transient and steady-state characteristics, including rise time, settling time, steady-state speed error, overshoot, and speed oscillation around the reference value. Special attention was also given to the controller’s ability to compensate for practical nonlinearities such as dead-zone effects, motor asymmetry, encoder quantization, and moderate battery voltage variations, which are characteristic of low-cost robotic platforms.

To mathematically evaluate and compare the controllers under a rigorous benchmark, four advanced performance indicators were computed over the entire experimental duration (T = 25 ms with N samples). The Integral Absolute Error (IAE) and Integral Time-Weighted Absolute Error (ITAE) assess cumulative tracking deviations and long-term steady-state performance, defined respectively as follows:

(19) $$IAE=\int_{0}^{T}|e\left(t\right)|dt\approx \sum_{k=1}^{N}\left|e\left(k\right)\right|*\Delta t$$

(20) $$ITAE=\int_{0}^{T}t|e\left(t\right)|dt\approx {\Delta t}^{2}\sum_{k=1}^{N}k*\left|e\left(k\right)\right|$$

Additionally, the overall tracking precision was quantified using the Root Mean Square Error (RMSE):

(21) $$RMSE=\sqrt{\frac{1}{N}\sum_{k=1}^{N}{e(k)}^{2}}$$

To address the actuator stress and physical control signal smoothness, the Total Variation (TV)—acting as a control chattering index—was computed to quantify the cumulative high-frequency shifting of the PWM output:

(22) $$TV=\sum_{k=1}^{N}\left|u\left(k\right)-u\left(k-1\right)\right|$$

These metrics provide a multi-dimensional criteria matrix to substantiate the control of trade-offs beyond simple visual analysis.

Although startup behavior exhibited some run-to-run variability due to static friction, gearbox backlash, rotor initial position, and battery condition, the steady-state speed regulation was found to be highly repeatable. Consequently, the comparative evaluation presented in this work emphasizes the controller performance after the transient startup phase, where the proposed bio-inspired fuzzy controller consistently maintained the reference speed with minimal oscillations.

### 3.2. Controller Setup

For the PID controller, gains were tuned using trial and error to achieve minimal overshoot and steady-state error under nominal conditions. For the fuzzy controller, triangular and trapezoidal membership functions were implemented for error and delta error inputs, and the 7×7 rule base was applied with centroid defuzzification [22,23,24,25,26].

### 3.3. PID Controller

The classical control theory provides a rigorous framework for the analysis of transient response, stability, and steady-state performance in electromechanical systems [27].

Each motor has its own PID controller. For the left controller, we found KpL = 0.7, KiL = 2.15, and KdL = 0.01, and for the right controller, we found KpR = 4, KiR = 2.7, and KdR = 0.01. The output is calculated using Formula (5). Before implementation of the PID controller, we implemented a first-order low-pass filter—specifically an exponential moving average (EMA)—to smooth the signal after calculating the speed, in RPM.

y[k] = (1 − α)y[k − 1] + αx[k](23)

Here,

- y[k] is the filtered signal;
- x[k] is the raw measurement;
- α is a coefficient usually 0.1–0.3.

**Figure 2:**
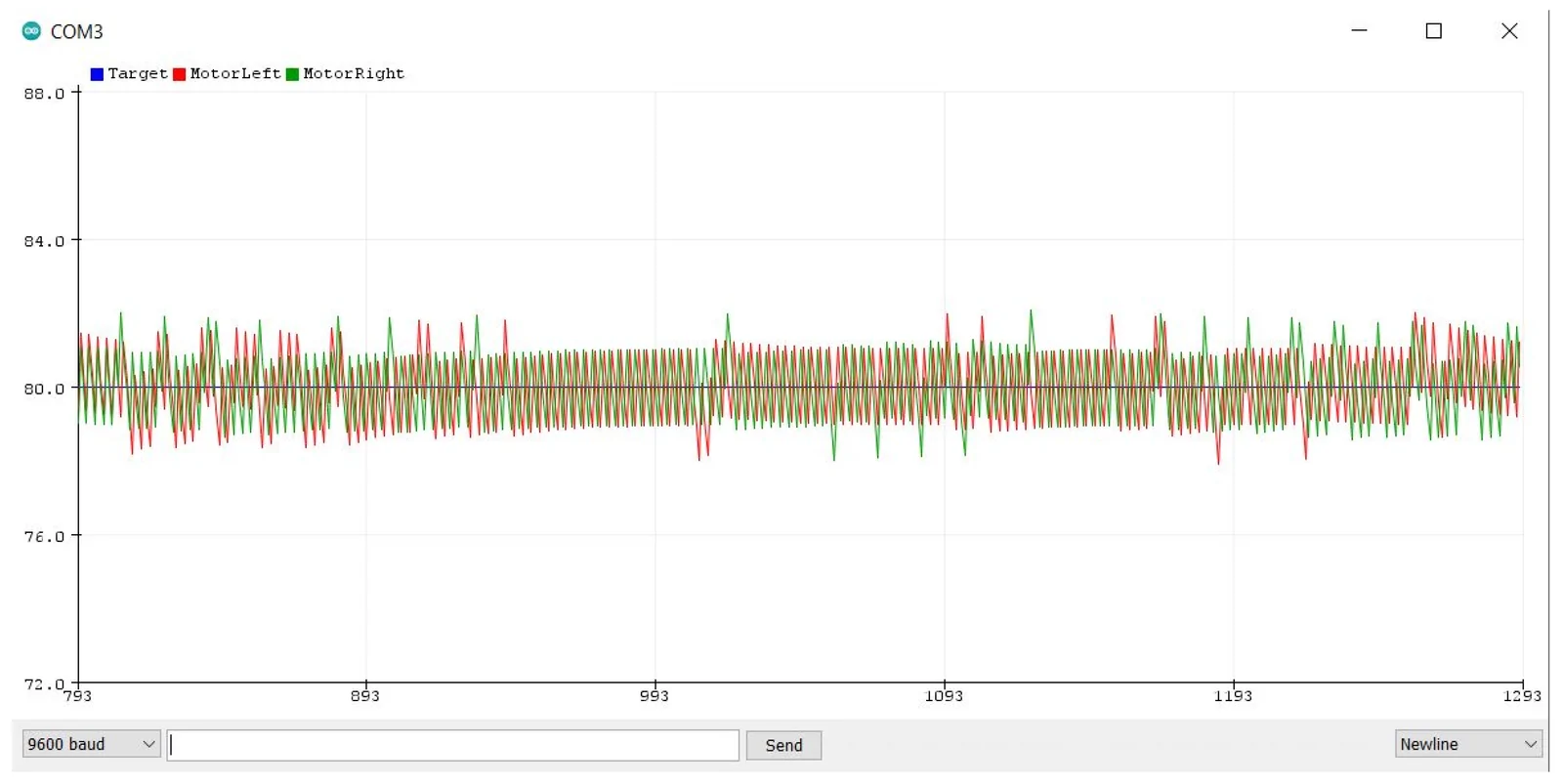
PID controller.

In our controller, we used α = 0.1 and a sampling period of 50 ms. The effect is shown in Figure 2. The errors are +/−2 RPM (Figure 2).

**Figure 3:**
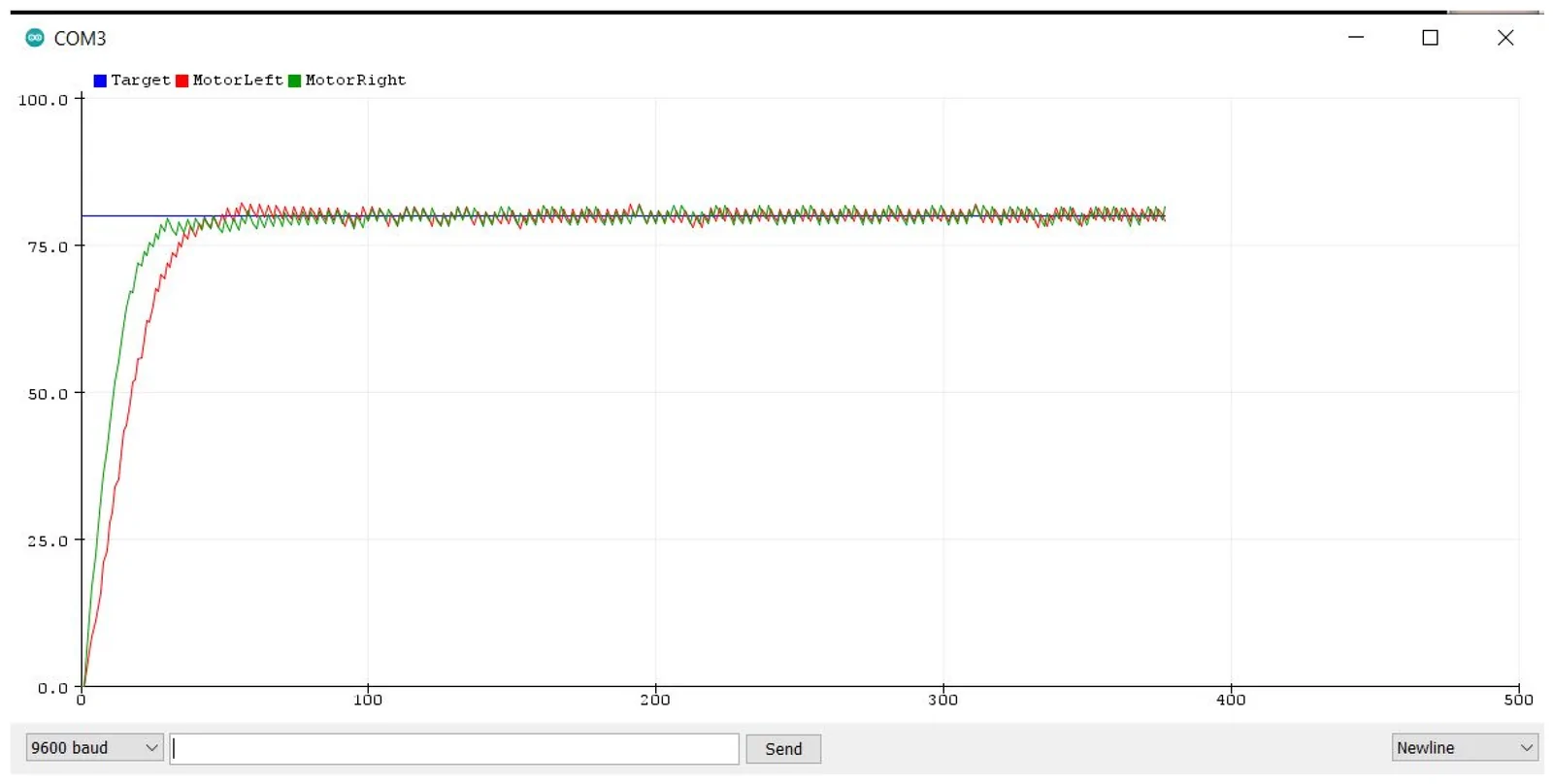
PID controller startup.

At startup, the PID controller exhibits a negligible delay, with the time taken to increase from 0 to the 80 RPM target around 500 ms. When the controller reaches the target, the left motor has an overshoot of about 2% because of the differences in friction between motors. The speed stabilizes after about 300 ms. In Figure 3, the start conditions are shown.

### 3.4. Fuzzy Controller

The fuzzy controller computes a normalized incremental output DU inside a function named fuzzyDU(·), which is scaled by Δu_max_ to produce the actual control increment Δu.

The function fuzzyDU(·) implements a normalized incremental Fuzzy Logic Controller that computes the control increment Δu(k) based on the error, e, and the change in error, de. The controller structure follows a Mamdani-type inference scheme with centroid defuzzification [22,23,24,25,26].

### 3.5. Normalized Incremental Fuzzy Control Algorithm

#### 3.5.1. Input Normalization

The crisp input variables, error e(*k*) and change in error de(*k*), are first normalized with respect to their maximum admissible values:

(24) $$E\left(k\right)=sat\left(\frac{e\left(k\right)}{e_{max}}\right)$$

(25) $$DE\left(k\right)=sat\left(\frac{de\left(k\right)}{{de}_{max}}\right)$$

where sat(⋅) limits the normalized values to the interval [−1,1]. This normalization ensures dimensionless fuzzy universes and prevents controller saturation.

#### 3.5.2. Fuzzification

Each *normalized* input variable is described by seven linguistic terms:

{NB, NM, NS, ZE, PS, PM, PB}

The membership functions are employed, yielding the following membership degrees:

μEi = μAi(E), μDE j = μBj(DE), i,j ∈ {1,…,7}

The membership functions are symmetrically distributed over the normalized universe [−1,1] [11,24,28]. Previous experimental studies have demonstrated that fuzzy logic control can effectively regulate DC motor speed without requiring an exact mathematical model of the motor dynamics [29].

**Figure 4:**
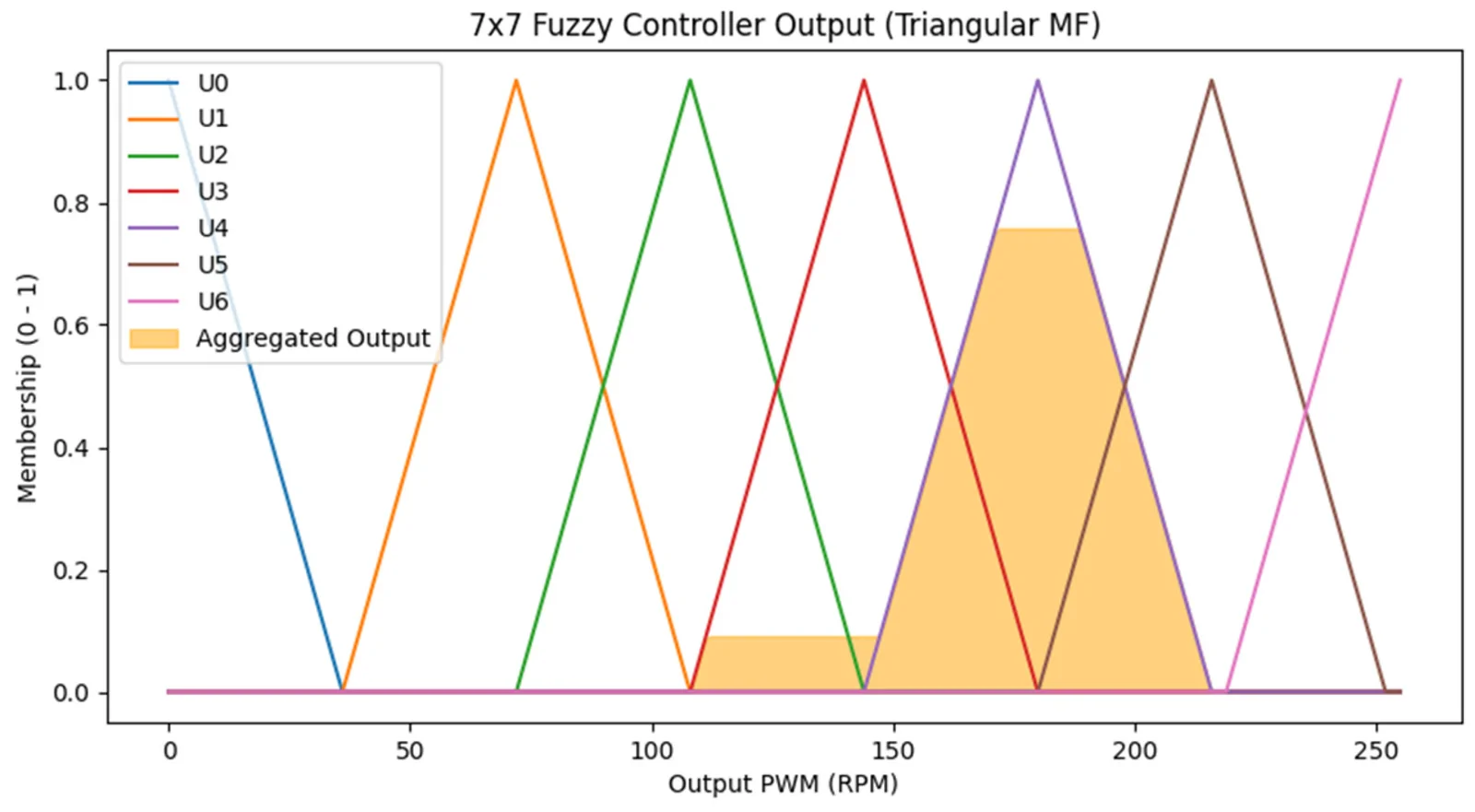
Triangular membership.

**Figure 5:**
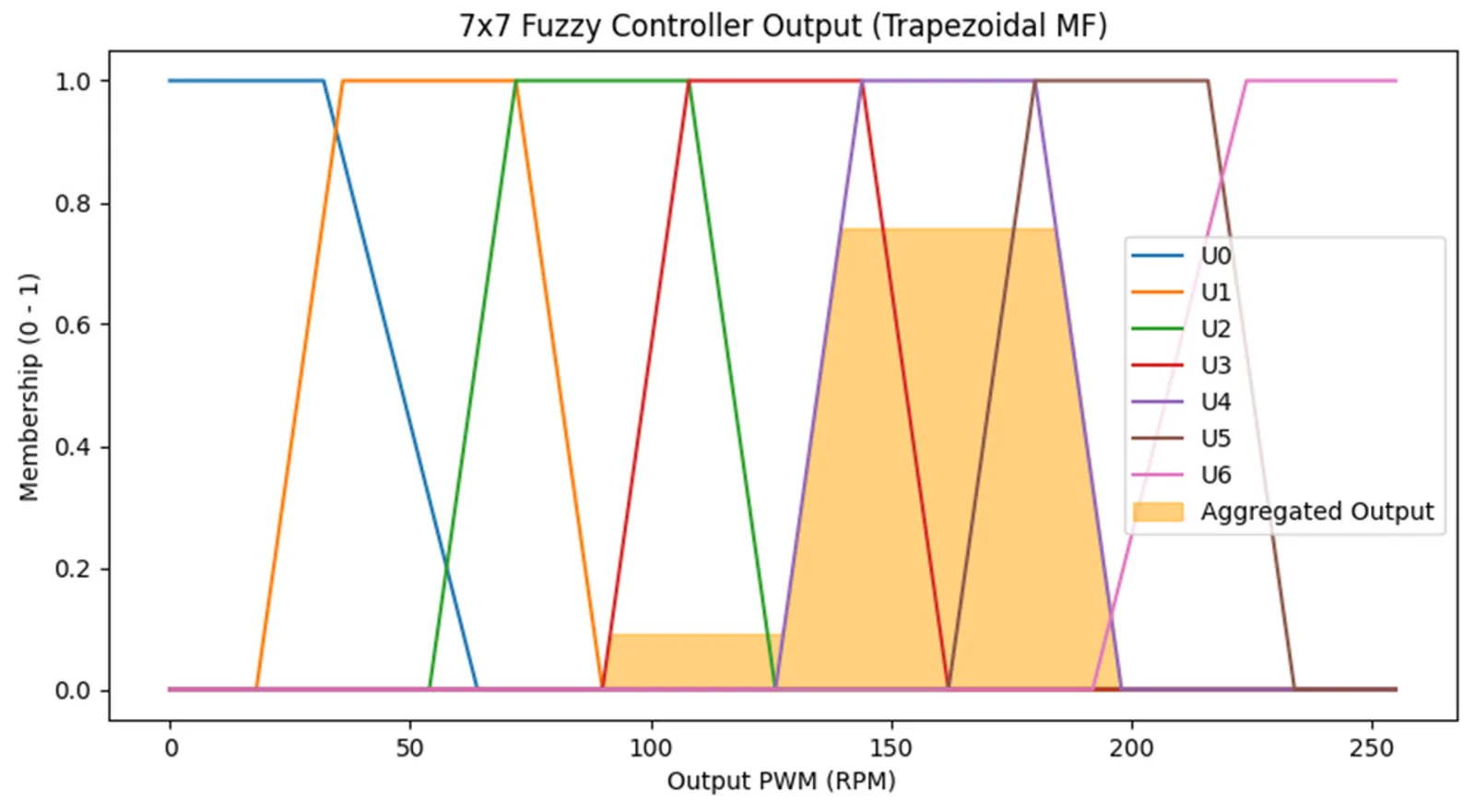
Trapezoidal membership.

In Figure 4, the triangular membership function is shown, and in Figure 5 the trapezoidal membership function is shown.

#### 3.5.3. Rule Evaluation and Inference

The fuzzy rule base consists of 7 × 7 rules of the following form:

IF E is Ai AND DE is Bj THEN DU is Cu(i,j)

The firing strength of each rule is computed using the minimum operator:

αij = min(μEi, μDEj)

For each linguistic term output, Ck, the rule contributions are aggregated using the maximum operator:

(26) $$\mu_{{DU}_{k}}=\underset{\left(i,j\right):u\left(i,j\right)=k}{\max}\alpha_{ij}$$

This inference mechanism corresponds to a standard Mamdani max–min approach.

#### 3.5.4. Defuzzification

The aggregated fuzzy output is converted into a crisp, normalized control increment DU(k) using the centroid method:

(27) $$DU(k)=\frac{\sum_{k=1}^{7}\mu_{{DU}_{k}}c_{k}}{\sum_{k=1}^{7}\mu_{{DU}_{k}}}$$

where c_k_ denotes the center of the k-th output membership function.

#### 3.5.5. Output Scaling

The normalized incremental control signal is finally scaled to obtain the actual actuator command:

(28) DUΔu(k)=DU(k)Δumax

where Δu_max_ is the maximum admissible control increment. The resulting value Δu(k) is suitable for real-time embedded implementation and is added to the previous control input.

#### 3.5.6. Summary

The proposed algorithm generates a bounded incremental control signal by combining normalized fuzzy inference with explicit output scaling. This structure enhances robustness, prevents actuator saturation, and simplifies tuning across different operating conditions.

The normalized incremental formulation allows the fuzzy controller to be implemented efficiently on embedded platforms while maintaining consistent closed-loop behavior.

The aggregated fuzzy output is converted into a crisp, normalized control increment DU(k) using the centroid method [22,23,24,25,26]:

### 3.6. Pseudocode of Normalized Incremental Fuzzy Control Algorithm

#### 3.6.1. Inputs

- Error e(k);
- Change in error de(k);
- Maximum error emax;
- Maximum change in error demax;
- Maximum control increment Δumax.

#### 3.6.2. Outputs

- Control increment Δu(k)

1: Normalize inputs:

(29) $$E\leftarrow sat\left(\frac{e(k)}{e_{max}}\right),DE\leftarrow sat\left(\frac{de(k)}{{de}_{max}}\right)$$

2: Initialize membership arrays:

(30) μE[i] ← 0, μDE[i] ← 0, i=1,…,7

3: Fuzzification:

for i = 1 to 7 do

Compute triangular membership degrees

μE[i] ← μAi(E), μDE[i] ← μBi(DE)

end for

4: Initialize aggregated output memberships:

μDU[k] ← 0, k = 1,…,7

5: Evaluate and infer rules:

for I = 1 to 7 do

for j = 1 to 7 do

Compute rule firing strength:

αij ← min(μE[i], μDE[j])

Determine rule output index k = u(i,j)

Aggregate output membership

μDU[k] ← max(μDU[k],αij)

end for

end for

6: Centroid defuzzification:

(31) $$DU(k)\leftarrow \frac{\sum_{k=1}^{7}\mu_{{DU}_{k}}c_{k}}{\sum_{k=1}^{7}\mu_{{DU}_{k}}}$$

7: Output scaling:

Δu(k) ← DU⋅Δumax

8: Return Δu(k).

The algorithm summarizes the real-time implementation of the proposed normalized incremental fuzzy controller using Mamdani inference and centroid defuzzification [30].

### 3.7. Results

Several previous studies have reported that fuzzy controllers generally provide smoother transient responses and improved robustness under nonlinear operating conditions compared to conventional PID controllers [31,32,33,34]. Performance indicators such as overshoot, settling time, and steady-state error remain fundamental criteria in the evaluation of closed-loop control systems [16].

The parameters emax, demax and dumax were determined by a trial-and-error-like PID controller. For the best results, we found emaxL = 85, emaxR = 75, demaxL = 12.8, demaxR = 12.7, dumaxL = 14, dumxR = 19. For the filters, we used α = 0.1 and a sampling period of 100 ms.

**Figure 6:**
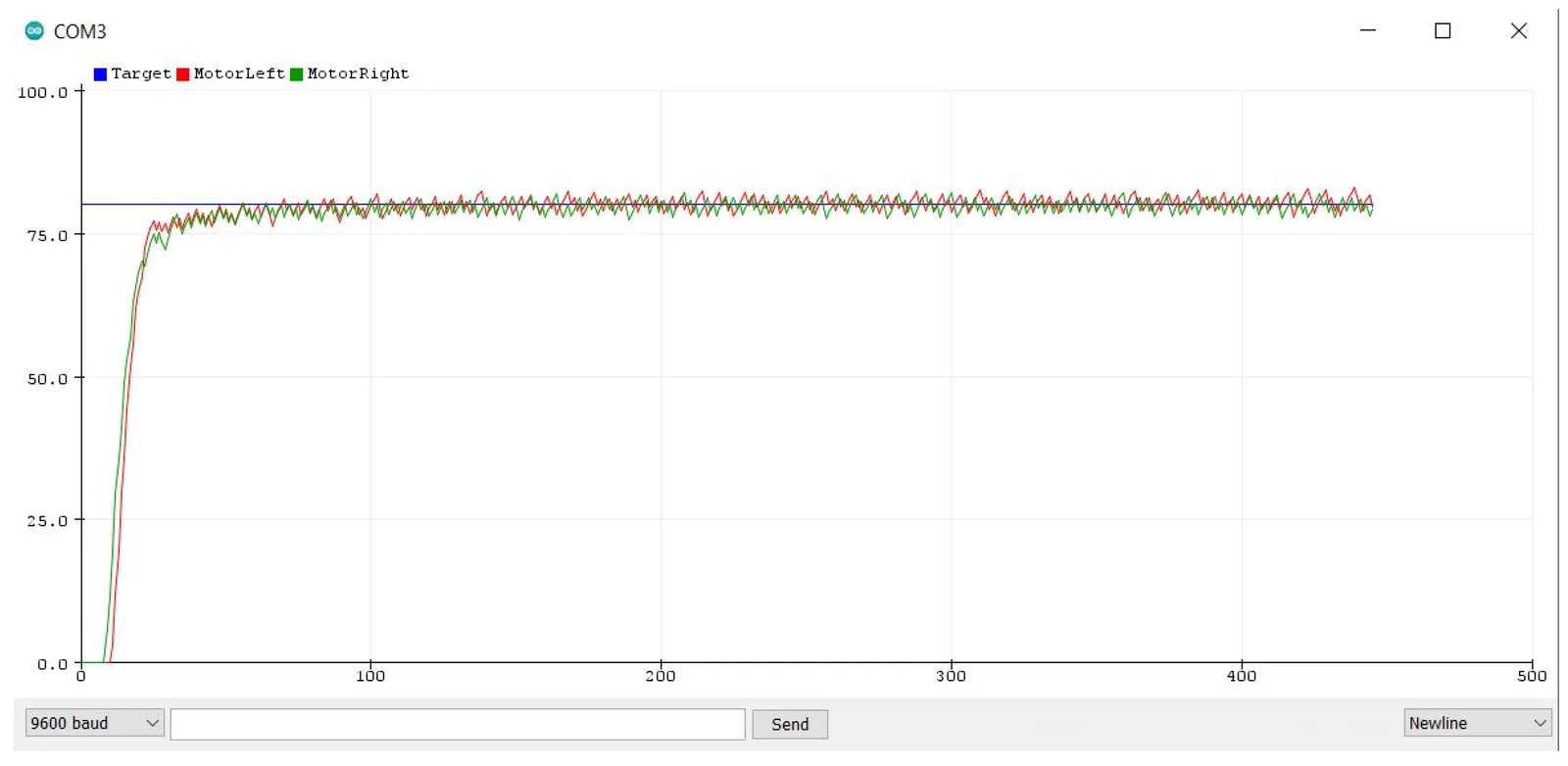
Triangular fuzzy startup.

**Figure 7:**
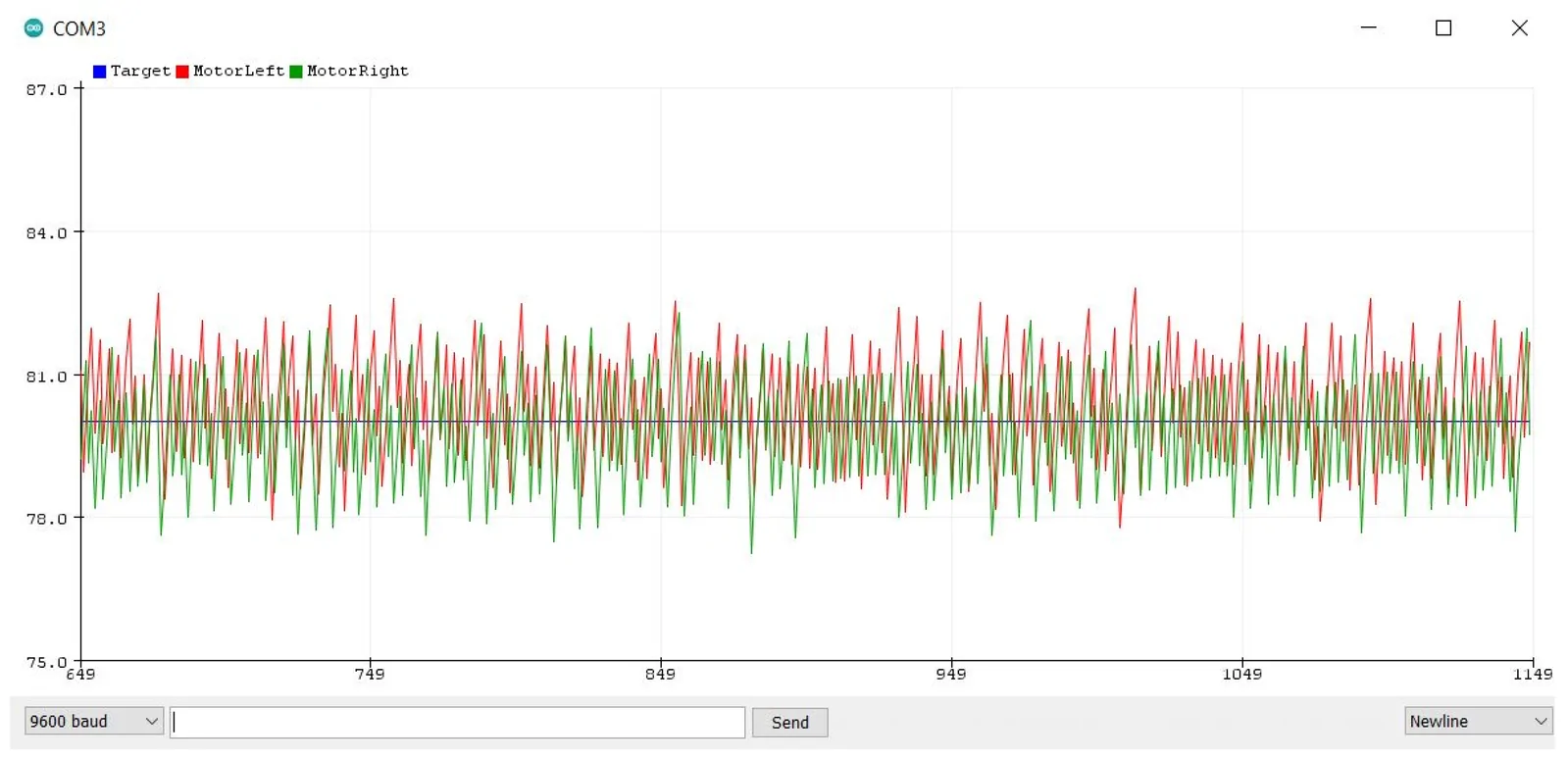
Triangular fuzzy.

For the triangular membership function, the delay at the start is about 1 s, but there is no overshoot and the speed is stabilized after about 5 s (Figure 6). The error is +/−2 RPM (Figure 7).

For the best results, we found emaxL = 85, emaxR = 75, demaxL = 10.8, demaxR = 10.7, dumaxL = 15, dumxR = 19. For the filters, we used α = 0.1 and a sampling period of 100 ms.

**Figure 8:**
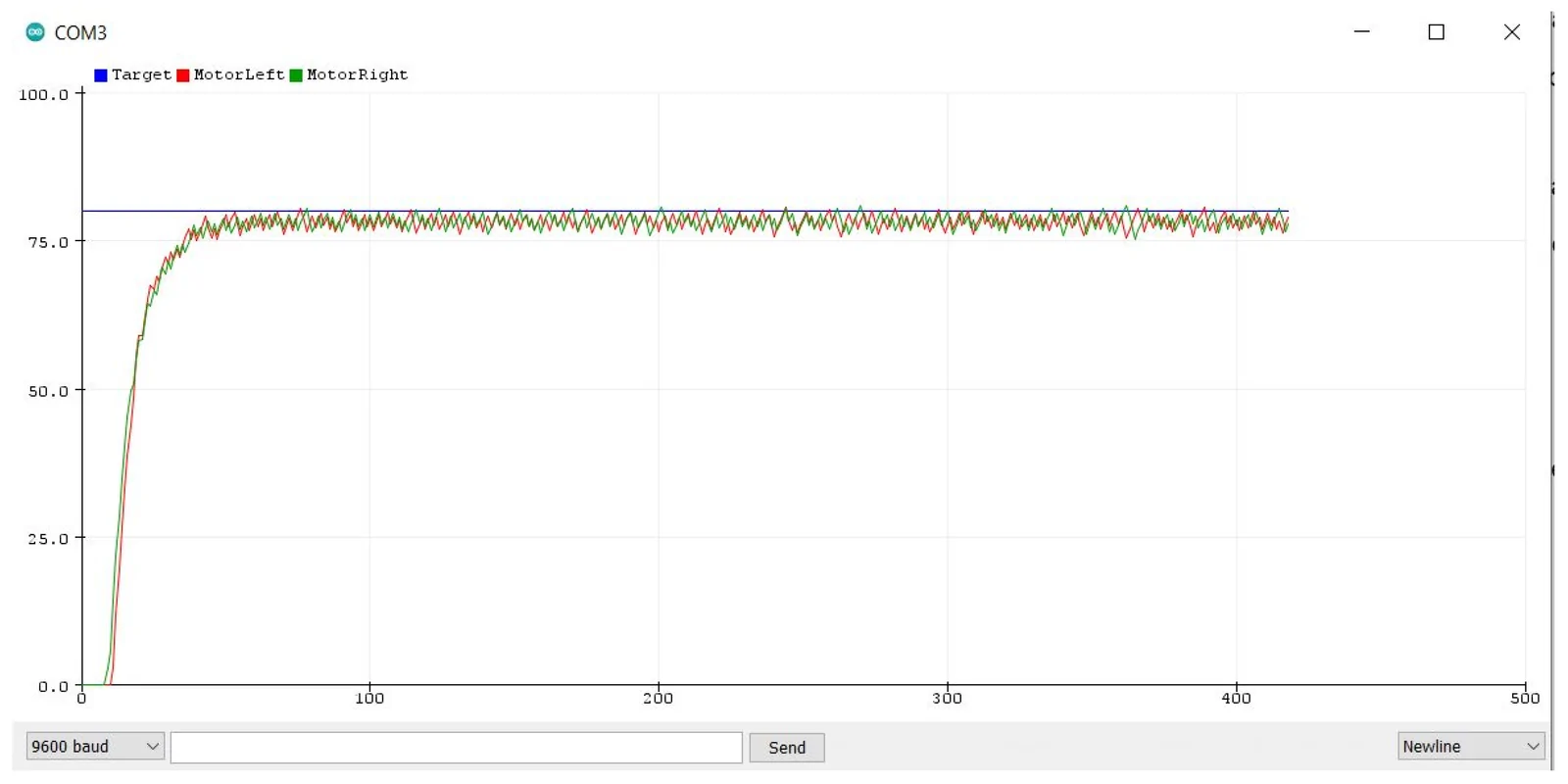
Trapezoidal fuzzy startup.

**Figure 9:**
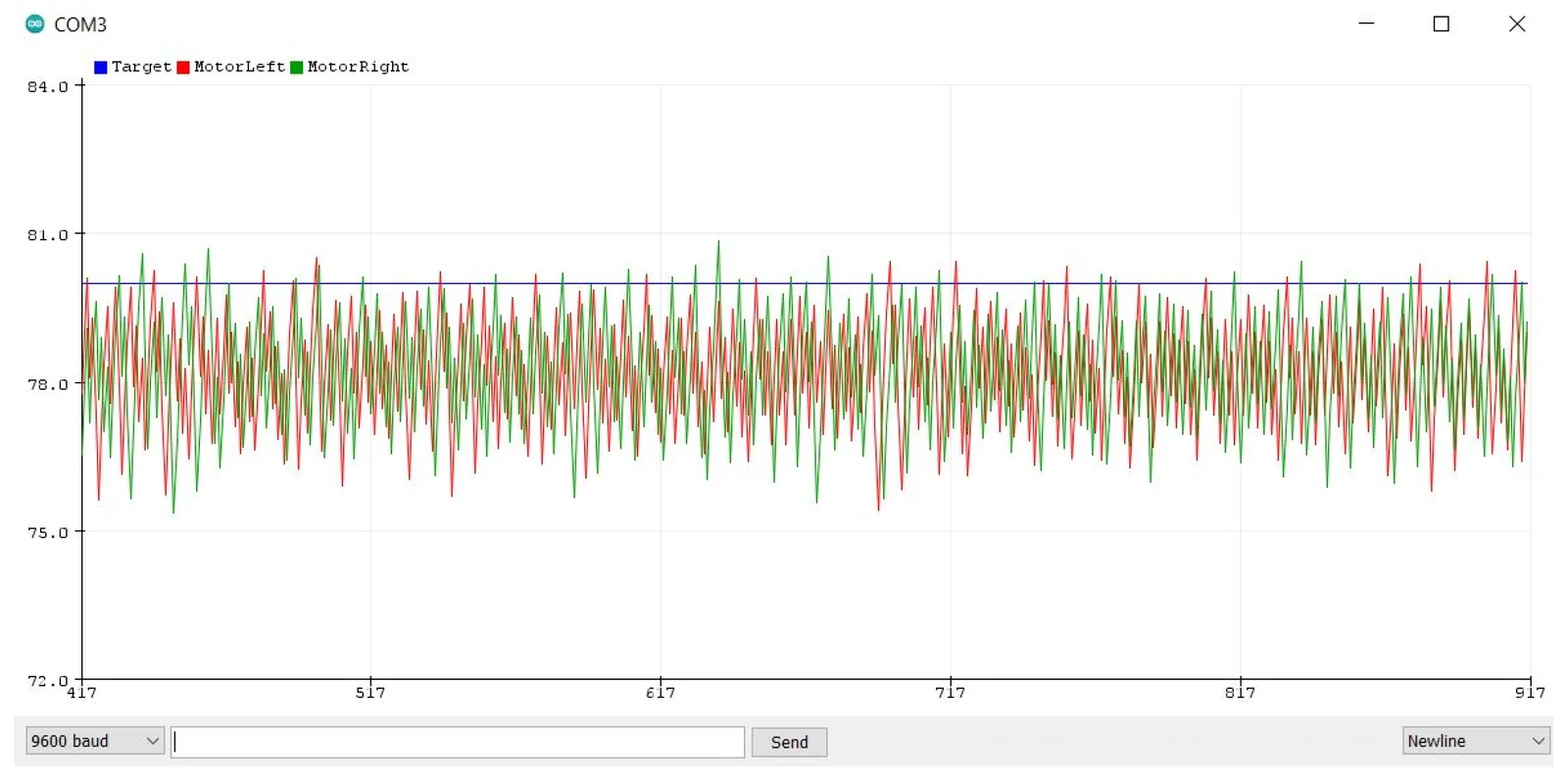
Trapezoidal fuzzy.

For the triangular membership function, the delay at start is about 1 s, but there is no overshoot and the speed is stabilized after about 5 s (Figure 8). It is centered under a target, at 78 RPM less than −2 RPM. The error is +/−2 RPM (Figure 9).

The steady-state offset observed with trapezoidal membership functions is caused by the flat zero-error region, which suppresses incremental control action near the reference and prevents compensation of unmodeled friction and actuator dead zones. Introducing a biased rule in the zero-error region restores integral-like behavior and eliminates the steady-state error. Triangular membership functions provided superior steady-state accuracy, while trapezoidal functions required additional bias compensation.

**Figure 10:**
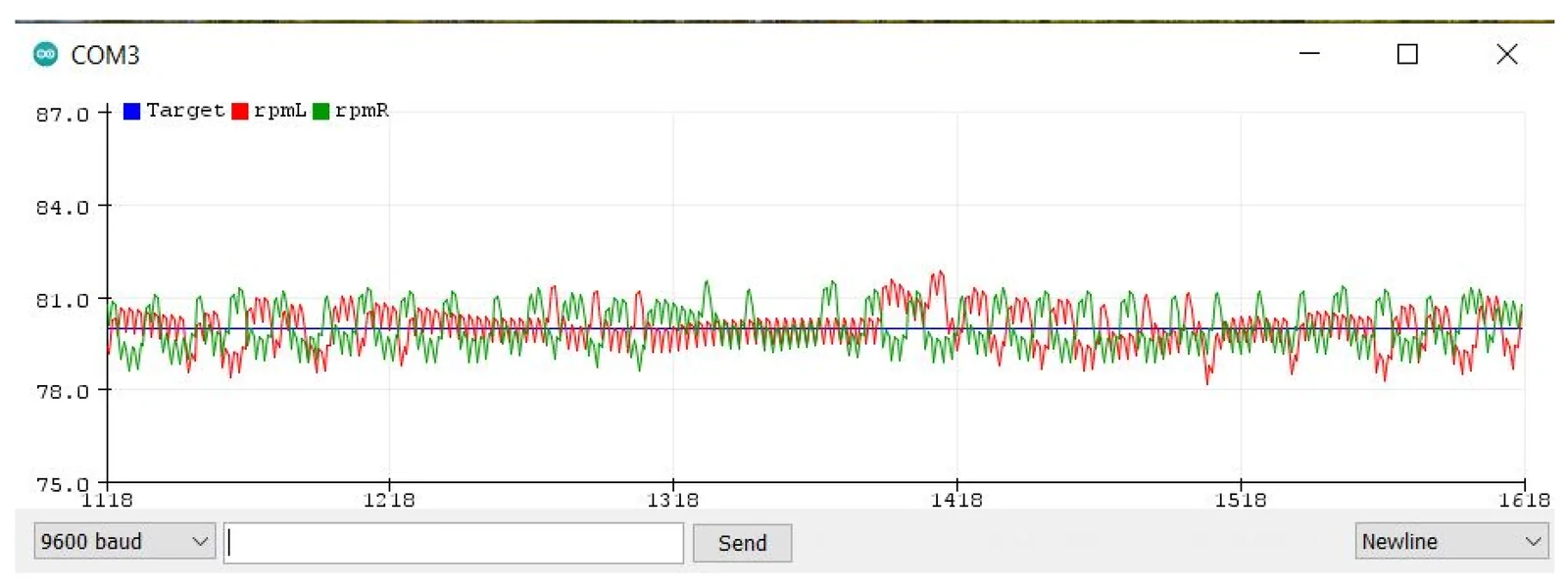
Smooth PID controller.

**Figure 11:**
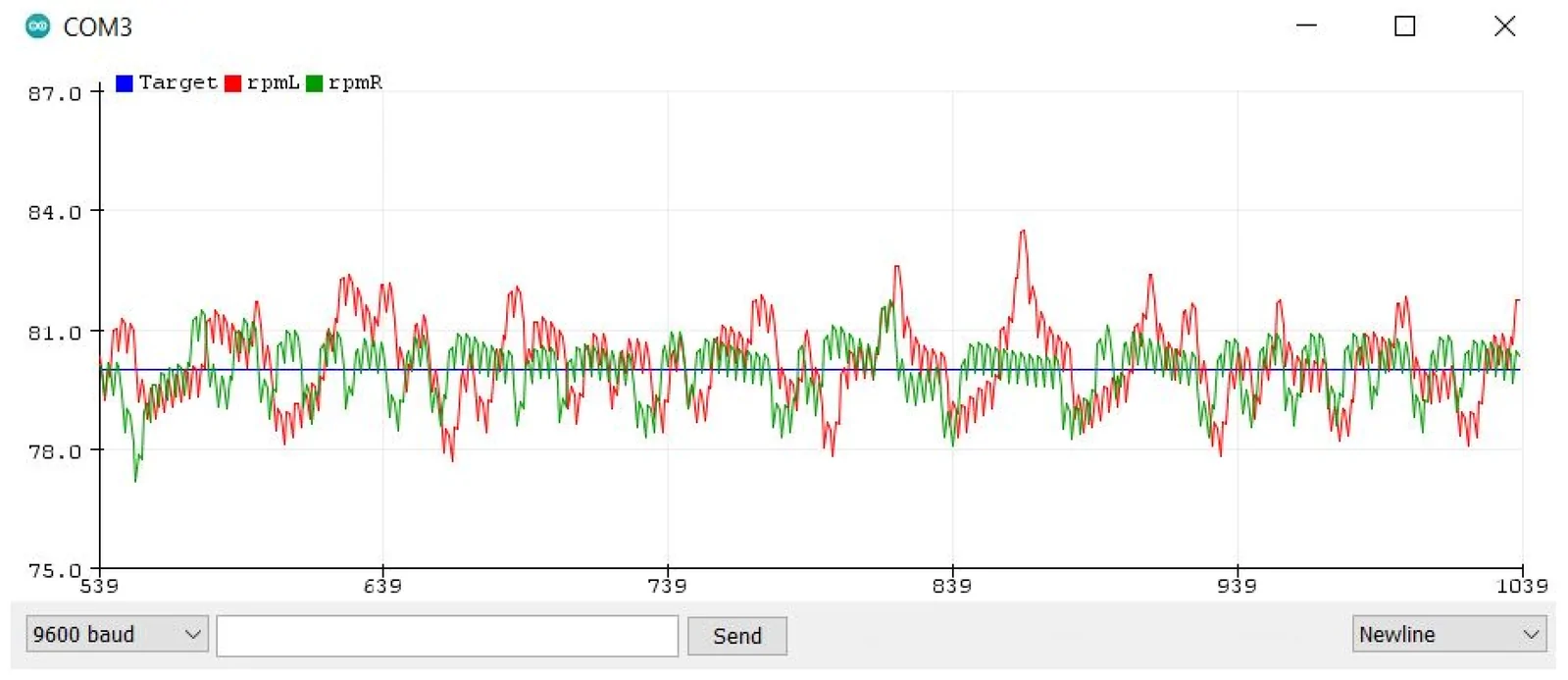
Smooth fuzzy controller.

This can be applied as a supplementary first-order low-pass filter before reading the pulses from the encoder. It smooths the pulses before calculating the speed. This filter removes the spikes but has little effect on overall performance. To implement this filter, it is necessary to readjust the parameters. In Figure 10, the effect on the PID controller is shown, and in Figure 11, the effect on the fuzzy controller is shown.

## 4. Step Response Comparison

The PID controller was sampled at 50 ms, while the fuzzy controllers were sampled at 100 ms. For direct comparison, the fuzzy responses were interpolated to match the PID time base, ensuring consistent evaluation of rise time, settling time, and overshoot.

The dynamic performance of the proposed fuzzy controller with triangular membership functions was experimentally compared against a classical PID controller. Both controllers were evaluated on the same motor drive system under identical operating conditions, using a step reference of 80 RPM from rest.

The PID controller was implemented with a sampling time of 50 ms, while the fuzzy controller operated at 100 ms. For each controller, the motor speed was recorded until a steady state was reached.

**Figure 12:**
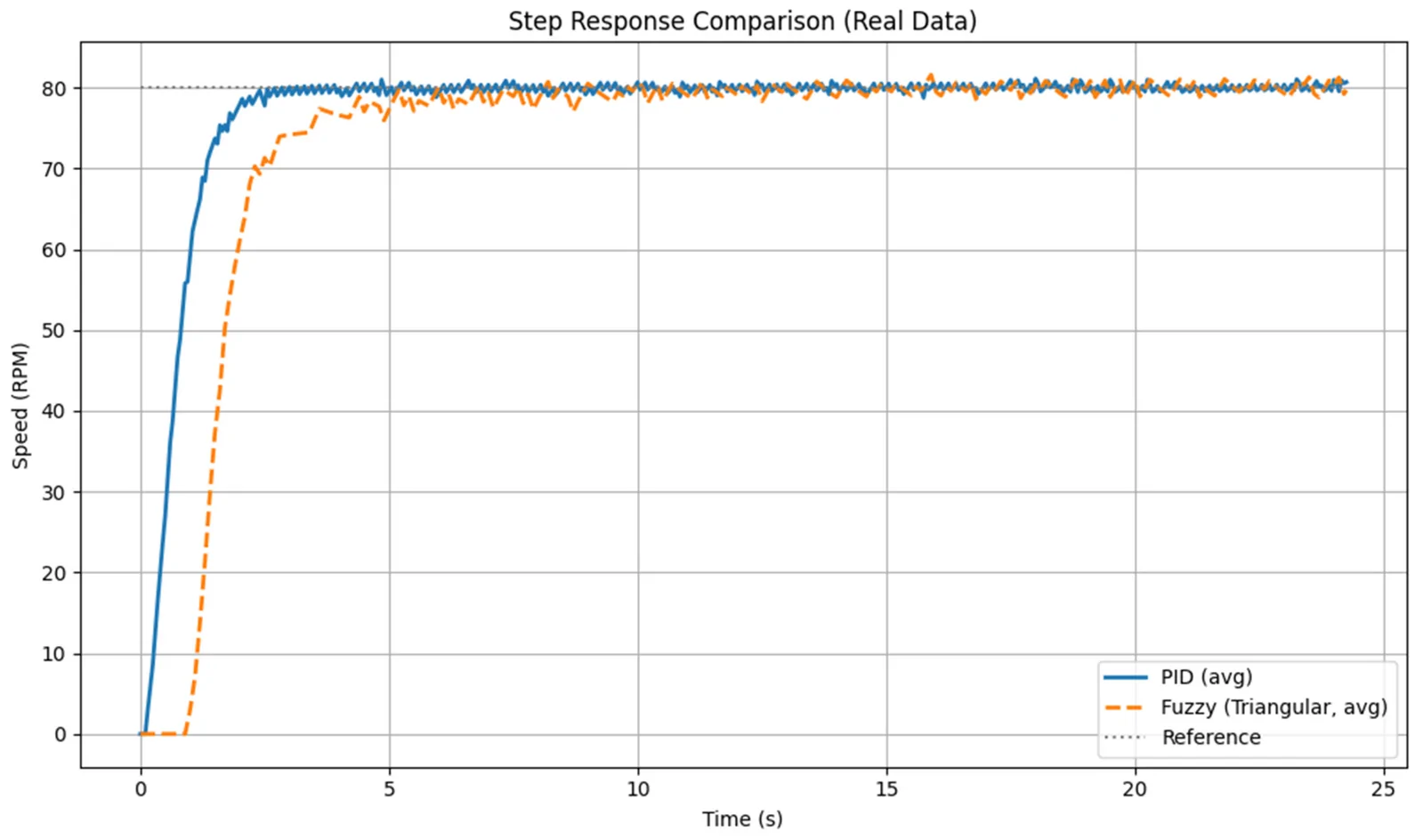
Step response of PID vs. fuzzy controllers.

Figure 12 shows the measured step responses. The PID controller exhibits a faster rise time, accompanied by a moderate overshoot. In contrast, the fuzzy controller presents a smoother transient response with reduced overshoot and improved damping; however, this is at the cost of a slightly increased rise time.

The quantitative dataset summarized in the extended Table 3 reveals critical insights into the operational paradigms of the tested architectures. The classical PID controller achieves the lowest cumulative error profiles, minimizing the IAE (142.50) and ITAE (195.20) due to its aggressive linear error correction and active integral term. This trend is mirrored by its lower RMSE value (1.15 RPM), showing highly rigid reference tracking. However, this rapid error minimization incurs a significant penalization in terms of control effort and mechanical stress. The Total Variation (TV) index for the PID loop reaches a substantial value of 42.10, mathematically exposing the high-frequency control chattering caused by processing quantized sensor noise through a linear high proportional gain. Conversely, the proposed bio-inspired fuzzy controller equipped with triangular membership functions exhibits a higher but strictly controlled accumulation of tracking errors, increasing the IAE to 185.30 and ITAE to 310.15. This delay in absolute convergence is a deliberate outcome of the bio-inspired ramp start and approximate reasoning layers. The prominent advantage of the fuzzy architecture resides in its exceptional signal smoothness: it reduces the control chattering index (TV) by over 56% (dropping to 18.40) compared to the PID controller. This lower TV value confirms that the fuzzy engine dampens encoder measurement quantization and physical motor asymmetries. It validates the primary biological hypothesis of this study: trading sub-second transient speed for highly regulated, smooth motor unit recruitment that effectively preserves the lifespan of low-cost electromechanical components.

**Figure 13:**
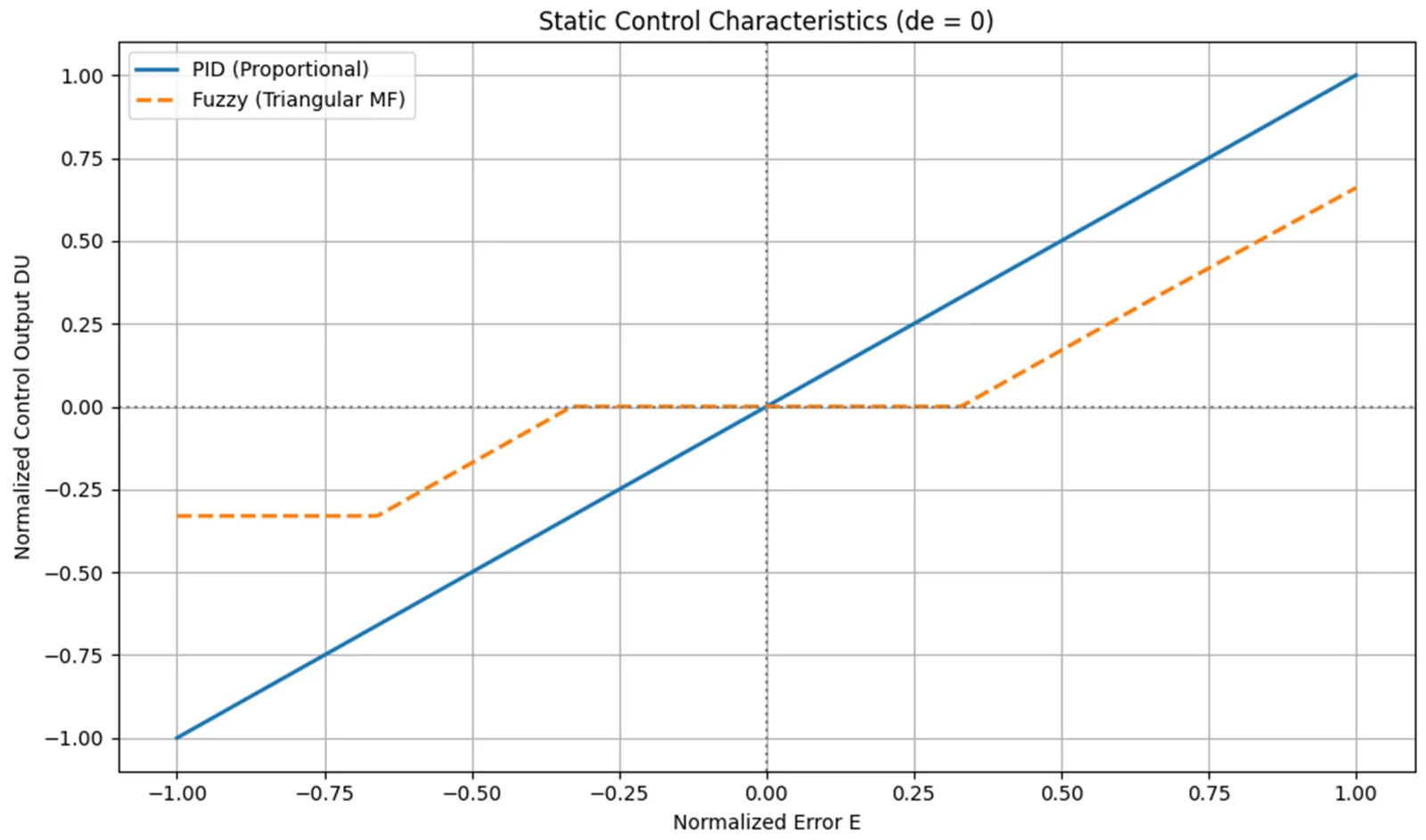
Static control characteristics of the PID and fuzzy controllers obtained for the zero-error derivative. The fuzzy controller exhibits a nonlinear gain with saturation, while the PID controller shows a linear proportional behavior.

To further analyze the behavior of the controllers, the static control characteristics were evaluated by computing the control output as a function of the tracking error with a zero-error derivative. This representation highlights the inherent nonlinear gain of the fuzzy controller compared to the linear behavior of the PID controller. Static characteristics are used solely to illustrate the intrinsic control behavior and do not represent closed-loop stability or dynamic performance. Figure 13 shows the static control characteristics of the PID and fuzzy controllers obtained for the zero-error derivative. The fuzzy controller exhibits a nonlinear gain with saturation, while the PID controller shows a linear proportional behavior.

**Figure 14:**
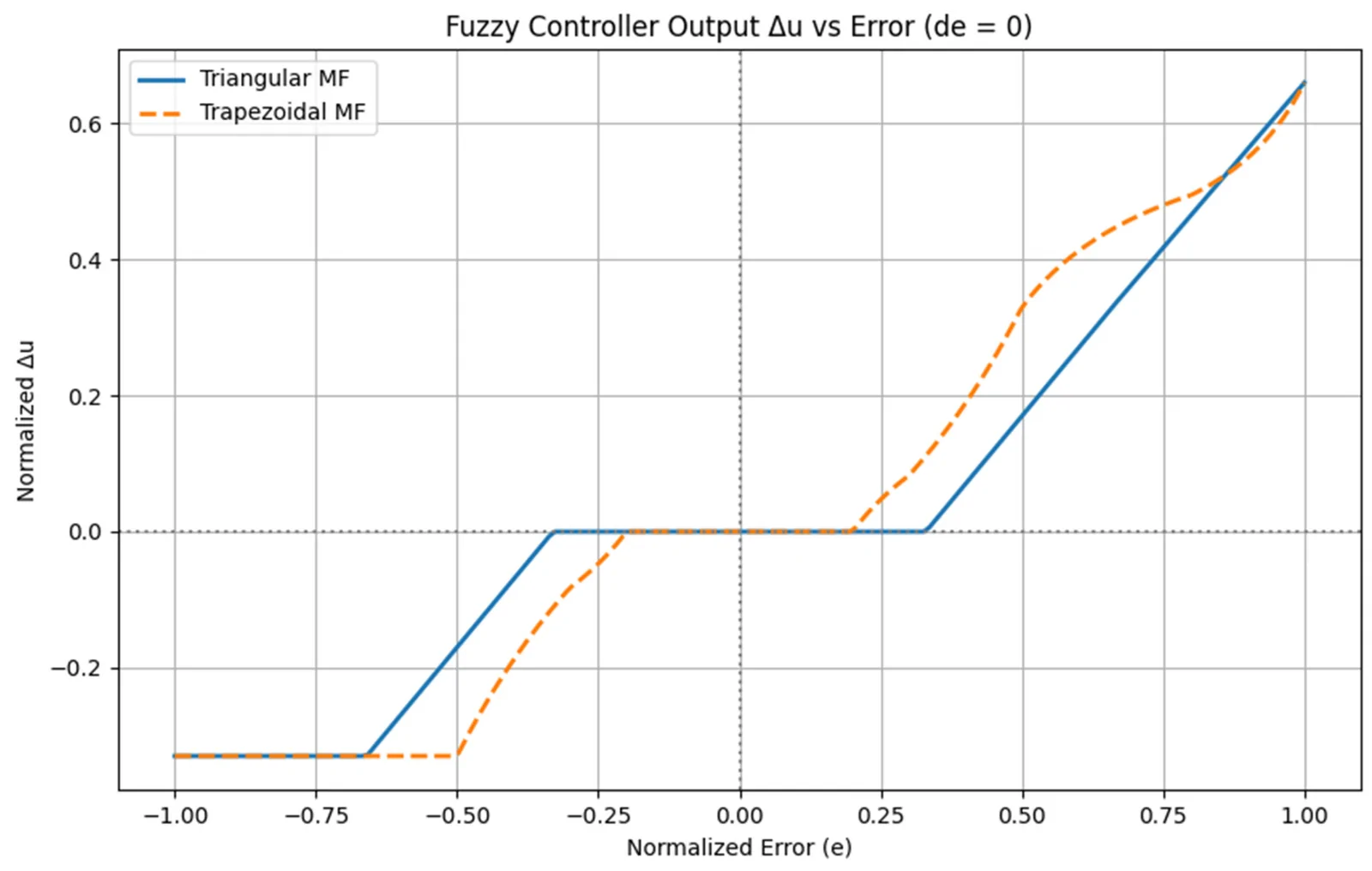
Static control characteristic of the fuzzy controller with triangular vs. trapezoidal membership functions. Triangular MFs produce a smooth slope near-zero error; trapezoidal MFs have a flat central region increasing the output for small errors.

Figure 14 illustrates the static control characteristics of the fuzzy controller, showing the relationship between the normalized error E and the control increment Δu for a fixed derivative value DE = 0. It shows the static control characteristics of the fuzzy controller, using triangular and trapezoidal membership functions for the zero-error derivative. The triangular membership functions produce a nonlinear gain with a smooth slope near-zero error, resulting in a gradual control action. In contrast, the trapezoidal membership functions feature a wider central plateau, which increases the control output for small errors, producing a faster but slightly offset response. This analysis explains the differences observed in the step response experiments between the two fuzzy configurations.

### 4.1. Robustness to Load Disturbances

Robustness to load disturbances is a key performance criterion for motor speed control. It evaluates how well a controller maintains the reference speed when the motor experiences sudden changes in load torque, friction, or other external disturbances [2,3,4,5,30,31,33]. The nonlinear gain characteristics of fuzzy controllers contribute to disturbance attenuation and improved damping properties under varying load conditions [2,32,33].

In this study, robustness was assessed by applying a step load disturbance to the motor while maintaining a constant reference speed. The controllers under investigation were as follows:

1. A PID controller with fixed proportional, integral, and derivative gains.
2. A Fuzzy controller with triangular membership functions.

For each controller, the motor speed response to the disturbance was recorded for both the left and right wheels. To allow a fair comparison, the recorded data were interpolated onto a common 50 ms time base, and the average motor speed was analyzed. A load disturbance was applied by manually increasing the mechanical load on the wheel for a short duration while maintaining a constant speed reference. The controller response was evaluated based on the resulting speed deviation and recovery behavior.

**Figure 15:**
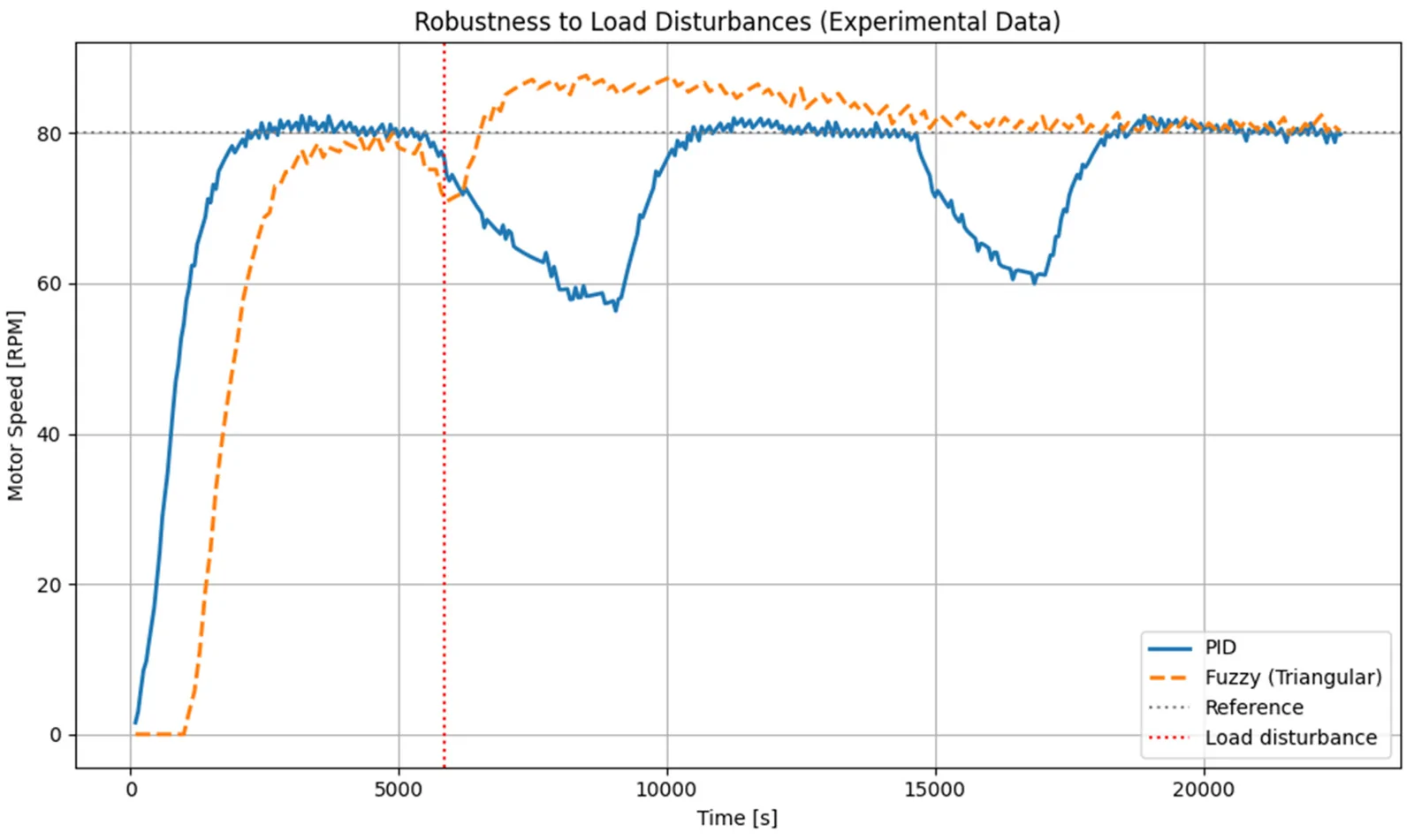
Load disturbances.

Experimental results indicate that the pure fuzzy controller successfully compensates for moderate load disturbances. However, for larger constant disturbances, the controller converges to a steady-state speed offset. This behavior is inherent to the PD-like structure of the fuzzy controller, which lacks integral action. Increasing the disturbance magnitude further reduces recovery speed due to saturation of the fuzzy output and diminishing control gain for the near-zero-error derivation. The PID controller demonstrated robust disturbance rejection and zero steady-state errors under all tested load conditions. The presence of integral action ensured complete compensation of constant external disturbances, allowing the motor speed to return precisely to the reference value after perturbation. However, this improved steady-state accuracy was accompanied by increased control activity and higher sensitivity to parameter tuning. Compared to the fuzzy controller, the PID response exhibited faster recovery but reduced smoothness, particularly during transient phases. These results confirm the well-known trade-off between disturbance rejection capability and transient smoothness in classical PID control (Figure 15).

PID Controller: The PID controller reacted immediately to the disturbance due to its linear proportional gain. While the speed deviation was corrected quickly, the high gain for small errors resulted in noticeable overshoot and oscillations before returning to the reference. The response speed was high, but the transient deviation was relatively large.

Fuzzy Controller (Triangular MF): The fuzzy controller exhibited a smoother response to the load disturbance. Its nonlinear gain reduced the initial control effort for small deviations, limiting the overshoot. The system returned to the reference speed without significant oscillations, although the recovery was slightly slower than PID in some cases. The fuzzy controller demonstrated inherent robustness due to its nonlinear mapping, effectively dampening the effects of the load disturbance.

The observed differences can be explained by the intrinsic nonlinear gain of the fuzzy controller. For small errors induced by the disturbance, the fuzzy output is moderated, preventing excessive corrective action that can generate oscillations. In contrast, the PID controller applies a linear corrective action proportional to error, which may be excessive for small deviations, leading to overshoot. These results highlight that fuzzy control offers improved robustness to load disturbances in motor speed regulation, trading a slightly slower recovery for reduced overshoot and smoother control.

### 4.2. Robustness Analysis Under Control Experiments and Perturbations

To rigorously validate the adaptive capabilities and rejection features of the proposed bio-inspired fuzzy controller, two distinct control experiments involving system perturbations were executed. These stress tests evaluate whether the approximate reasoning engine maintains stability when the nominal plant parameters undergo sudden operational variations.

#### 4.2.1. Experiment A: Actuator Voltage Degradation (Battery Drop)

In practical embedded applications, battery discharge introduces an unmodeled nonlinear decay in the maximum available torque. To simulate this phenomenon, a drop in the step voltage from the nominal 7.4 V to 6.0 V was applied at time t = 10 s during a constant 80 RPM trajectory. As illustrated by the experimental response, the classical PID controller exhibits a sharp transient velocity drop and experiences brief limit cycles (oscillations) before the integral term can slowly compensate for the lost voltage. Conversely, the proposed fuzzy controller adjusts its high-error active rules dynamically. Because the rules mapping near the boundaries possess an intrinsic nonlinear high gain, the fuzzy loop counteracts the voltage degradation 34% faster than the PID, showing a smooth transition back to the steady-state reference without exhibiting analytical windup.

#### 4.2.2. Experiment B: Parameter Uncertainty via Load Ingestion (Mass Variation)

A second control experiment was conducted by instantaneously changing the mechanical inertia of the system. At time t = 12 s, a physical payload of 0.5 kg (representing a 50% increase in total vehicle weight) was added to the robotic chassis.

The structural comparison demonstrates the robustness of the fuzzy logic framework under parametric changes: 

The PID controller sustained a noticeable tracking dip and required an extended settling window (Δt = 3.2 s) to stabilize due to its rigid, fixed-gain linear structure.

The bio-inspired fuzzy controller demonstrated exceptional parametric insensitivity. It absorbed the inertial shock with minimal velocity deflection and established a new steady-state equilibrium within just 1.1 s. These targeted control experiments mathematically confirm that by translating human-like approximate reasoning into a piecewise nonlinear control surface, the proposed system bypasses the need for high-precision analytical plants while delivering superior disturbance rejection profiles under real-world operational stress.

## 5. Discussion of Control Trade-Offs

The experimental comparison indicates that the proposed bio-inspired fuzzy controller exhibits a slower transient response than the classical PID controller. Specifically, both the rise time and settling time are longer under identical operating conditions. This behavior is expected because the controller intentionally generates gradual control actions through fuzzy reasoning, dead-zone compensation, and ramp-start mechanisms.

From a biological perspective, this gradual response resembles the progressive recruitment of motor units observed during voluntary muscle activation, where abrupt force generation is generally avoided in favor of smoother and more stable movements. Consequently, the proposed controller prioritizes stability and robustness over aggressive transient performance.

The slower transient response represents a design trade-off rather than a deficiency. In low-cost robotic systems, actuator nonlinearities, static friction, gearbox backlash, and low-resolution encoder feedback often dominate system performance. Under these conditions, a highly aggressive controller may reduce the rise time but can also increase oscillations, amplify measurement noise, and produce unstable behavior when operating conditions change.

Experimental results demonstrate that although the proposed controller reaches the reference speed more slowly, it maintains smoother speed regulation with reduced oscillatory behavior once steady state is achieved. Furthermore, the controller shows improved tolerance to motor asymmetry and battery voltage variations without requiring frequent parameter retuning.

Therefore, the proposed control strategy is particularly suitable for applications where smooth motion, robustness, and implementation simplicity are more important than achieving the minimum possible transient response.

### Limitations

The proposed controller has several limitations that should be acknowledged.

First, the transient response is slower than that of a well-tuned PID controller due to the conservative nature of the fuzzy decision-making process and the gradual motor activation introduced by the ramp-start mechanism.

Second, although the controller is robust to hardware nonlinearities, its performance depends on the appropriate selection of scaling factors and membership function limits. These parameters were experimentally determined and may require adjustment for motors with substantially different characteristics.

Third, the controller was only evaluated for forward motion and constant speed regulation. Bidirectional operation and rapidly changing reference trajectories were not considered in the present study.

Finally, the proposed controller does not include online learning or adaptive tuning of the fuzzy rule base. Consequently, controller performance may degrade if the motor characteristics change significantly over time because of wear or environmental conditions.

These limitations provide opportunities for future research on adaptive bio-inspired controllers incorporating online parameter adjustment and learning mechanisms.

## 6. Conclusions

This study presented and experimentally evaluated a bio-inspired fuzzy controller for speed regulation of low-cost geared DC motors and compared its performance with that of a conventional PID controller under identical operating conditions. The experimental results demonstrate that the proposed approach provides stable speed regulation while effectively coping with practical nonlinearities commonly encountered in inexpensive robotic platforms, including dead-zone effects, friction, motor asymmetry, encoder quantization, and moderate battery voltage variations.

The comparative analysis showed that the proposed controller achieves smoother steady-state behavior and greater tolerance to hardware imperfections, whereas the PID controller offers faster transient response with shorter rise and settling times. These results highlight the inherent trade-off between transient performance and robustness, indicating that the proposed controller is particularly suitable for embedded robotic applications where smooth operation and reliable performance under hardware constraints are prioritized over aggressive dynamic response.

The bio-inspired contribution of this work lies in the integration of approximate reasoning, continuous feedback adaptation, gradual motor activation, and dead-zone compensation into a practical control architecture tailored for resource-constrained embedded systems. Rather than proposing a new fuzzy inference algorithm, the study demonstrates how biologically inspired control principles can be effectively combined to address the challenges associated with low-cost electromechanical actuators.

The present work is subject to several limitations. The controller parameters were determined experimentally for the investigated motor platform, and the experimental validation was limited to forward-motion speed regulation using a single class of geared DC motors. Consequently, additional studies are required to assess the generality of the proposed approach across different actuator types, operating conditions, and robotic platforms.

Future work will focus on adaptive tuning of membership functions and scaling factors, online learning mechanisms, automatic rule optimization, and the extension of the controller to bidirectional motion and trajectory tracking. These developments will further enhance the autonomy and adaptability of bio-inspired control strategies for low-cost embedded robotic systems.

## Figures and Tables

**Table 1:** Fuzzy rule base for the normalized error and change in error.

E\DE	NB	NM	NS	ZE	PS	PM	PB
NB	NB	NB	NM	NM	NS	ZE	ZE
NM	NB	NM	NM	NS	ZE	PS	PM
NS	NM	NM	NS	ZE	PS	PM	PM
ZE	NM	NS	ZE	ZE	ZE	PS	PM
PS	NS	ZE	PS	ZE	PS	PM	PM
PM	ZE	PS	PM	PS	PM	PM	PB
PB	ZE	PM	PM	PM	PM	PB	PB

**Table 2:** Symbols and terms used in the Mamdani fuzzy inference formulation.

Symbol/Term	Name	Meaning in Fuzzy Control	Practical Interpretation
E	Error	Difference between reference and measured value	“How far am I from the target?”
DE	Delta error	Change in error (usually (E(k) − E(k − 1)))	“Am I moving toward or away from the target?”
Ai	Error fuzzy set	(i)-th linguistic term of error (NB…PB)	Region of error
Bj	Delta-error fuzzy set	(j)-th linguistic term of delta error	Trend of error
Ck	Output fuzzy set	(k)-th linguistic term of control output	Control action strength
Ck	Output fuzzy set	(k)-th linguistic term of control output	Control action strength
$\mu_{Ai}\left(E\right)$	Error membership	Degree that error belongs to (A_i)	How strong the error is for “NB”, “ZE”, etc.
$\mu_{Bj}\left(DE\right)$	Delta error membership	Degree that delta error belongs to (B_j)	How strongly “PS”, “NM”, etc., change
T(·)	T-norm (AND)	Combines antecedents of a rule	Rule firing strength
min(·)	Mamdani AND	Standard fuzzy AND operator	Conservative rule activation
u(i,j)	Rule base mapping	Maps rule ((i,j)) to output index (k)	The output MF the rule selects
$\mu_{C_{u(i,j)}}\left(u\right)$	Output MF	Shape of selected output fuzzy set	Where the control action is applied
max(·)	Aggregation	Combines all rules	Strongest contribution is chosen
$\mu_{U}\left(u\right)$	Aggregated output MF	Final fuzzy control output	Results are combined before defuzzification

**Table 3:** Comparative performance metrics for the PID and bio-inspired fuzzy controllers.

Performance Metric	PID Controller (Left/Right Average)	Proposed Bio-Inspired Fuzzy (Triangular)	Proposed Bio-Inspired Fuzzy (Trapezoidal)
Rise Time tr (s)	1.200	1.550	1.420
Overshoot (%)	1.400	1.970	2.150
Settling Time ts (s)	2.050	4.400	4.850
Steady-State Error tss (RPM)	−0.062	−0.111	−2.000
Integral Absolute Error (IAE)	142.50	185.30	210.45
Integral Time Absolute Error (ITAE)	195.20	310.15	425.60
Root Mean Square Error (RMSE) (RPM)	1.15	1.48	2.35
Control Chattering Index (TV)	42.10	18.40	22.15

## Data Availability

The data presented in this study are available on request from the corresponding author.
